# The use of posteromedial portal for arthroscopic treatment of synovial chondromatosis of the knee: a case report

**DOI:** 10.1186/s13256-022-03667-2

**Published:** 2022-12-10

**Authors:** Luigi Zanna, Gregorio Secci, Matteo Innocenti, Niccolò Giabbani, Roberto Civinini, Fabrizio Matassi

**Affiliations:** Orthopaedic Clinic CTO, University of Florence, Largo Palagi 1, 50139 Florence, Italy

**Keywords:** Synovial chondromatosis, Arthroscopic surgery, Posterior compartment of the knee, Posteromedial portal, Case report

## Abstract

**Background:**

The synovial chondromatosis is an uncommon proliferative metaplastic process of the synovial cells that can develop in any synovial joint. An isolated primary chondromatosis of the posterior compartment of the knee is uncommon and few cases are reported in literature. Our purpose is to describe a rare case of primary chondromatosis of the knee posterior compartment and report the arthroscopic loose bodies excision through a difficult posteromedial portal, avoiding the use of the accessory posterior portal, most commonly reported for approaching this disease.

**Case presentation:**

We report a rare case of a 35-year-old Caucasian male patient with diagnosis of chondromatosis of the posterior knee compartment. The radiographs showed multiple loose bodies of the posterior compartment. The MRI revealed minimal synovial hypertrophy areas, multiple osteophytes in the intercondylar notch, and loose bodies in the posteromedial compartment. The CT allowed us to assess the bony structures, the morphology of the intercondylar notch, and the presence osteophytes of the medial and lateral femoral condyles. The CT images were crucial to plan how to reach the posterior compartments of the knee through a trans-notch passage. The patient underwent arthroscopic surgery using anteromedial, anterolateral, and posteromedial portals. The tunneling through the intercondylar osteophytes was performed to allow the arthroscope to pass trans-notch. To avoid additional accessory posterior portals, we used a 70° arthroscope to better explore the posterior knee compartment. The cartilage-like bodies were removed and synovectomy of the inflamed areas was performed. The clinical and radiological follow-up was 12 months and the patient showed excellent clinical outcomes, returning to his activities of daily living and sport activity.

**Conclusion:**

Our case report highlights the importance of the arthroscopic approach to treat synovial chondromatosis, despite the involvement of the posterior compartment of the knee. An optimal preoperative imaging allows to plan for the proper surgical procedure even in patients with severe osteoarthritis. Moreover, the adoption of an intercondylar notch tunneling and a 70° arthroscope can help surgeons to better explore the posterior knee compartment, avoiding an accessory posterior trans-septal portal. Therefore, a synovectomy of the inflamed foci may be useful to prevent recurrence.

## Background

Synovial chondromatosis (SC), also known as Reichel syndrome [1], is an uncommon proliferative metaplastic process of the synovial cells that can affect any synovial joint. It is characterized by proliferation of chondrocytes and transformation of the synovia to a well-differentiated cartilage or osteochondral nodules that could detach from joint surface, creating loose bodies. It is usually a monoarticular disease, involving large joints such as the hip, shoulder, and mainly the knee [2, 3]. Owing to the metaplastic nature of SC, some authors reported a small risk of transformation to chondrosarcoma [4]. SC diagnosis and treatment at early phase is crucial to prevent the occurrence of secondary osteoarthritis and malignant transformation, as well as to avoid the invalidating sequelae. Primary SC commonly occurs in ages ranging from 20 to 50 years [5], and twice more frequently in men [6]. Secondary SC results from diseases that lead to synovitis and joint destruction, such as traumatic injury, infections, and neuropathic osteoarthropathy, particularly in patients ranging from 50 to 60 years [3]. According to Milgram [7], SC may be classified in three stages on the basis of gross and pathological findings. Clinical presentation is nonspecific, and may include diffuse articular pain, stiffness, swelling, and crepitus with symptoms of locking. The diagnosis is often made by a detailed anamnesis, physical examination, radiographs, computed tomography (CT), and magnetic resonance imaging (MRI). The treatment of SC may be conservative for mild forms, but in the presence of locking symptoms or decreased range of motion (ROM), surgery is the gold standard treatment [3]. Surgery can be performed with an open or arthroscopic approach. The arthroscopy has gradually replaced a traditional open approach [8], resulting in low morbidity, low postoperative pain, better cosmetic results, early recovery of range of motion, short rehabilitation course, and an early return to previous function [9]. Of note, SC commonly affects the anterior compartment of the knee [9], and only a few cases of isolated primary SC of the posterior compartment are reported in literature [10–12]. The posterior part of the knee is divided into the posteromedial and posterolateral sites by a septum which make this space through not readily accessible through conventional anterior arthroscopic portals [12]. The aim of our work is to describe a rare case of primary SC of the posterior compartment of the knee in a young patient with a high grade of osteoarthritis. We report the procedure of arthroscopic loose bodies excision by the use of posteromedial portal through a complex trans-notch passage, without additional accessory posterior portals, most commonly adopted in the surgical management of SC of the posterior compartment of knee.

## Case presentation

**Fig. 1:**
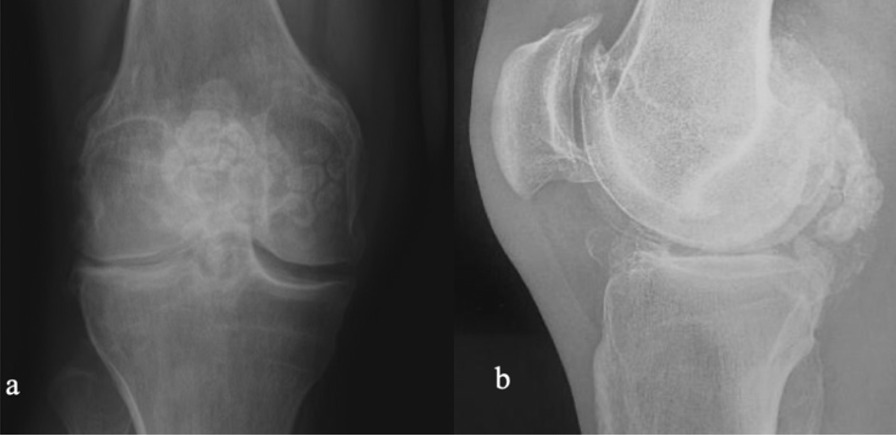
**a** Anterior–posterior and **b** lateral radiographs of the right knee. Multiple loose bodies in the posterior compartment

**Fig. 2:**
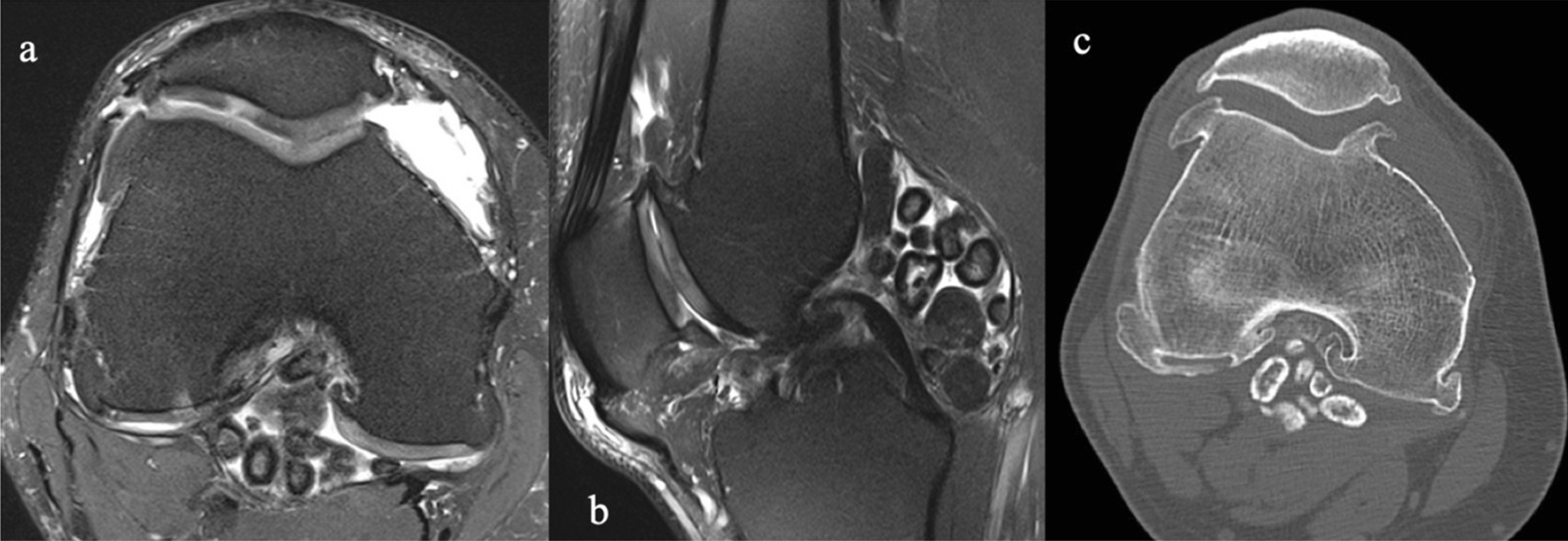
**a** MRI axial view and **b** MRI sagittal view. Chondropathy, minimal synovial hypertrophy areas, multiple osteophytes in the intercondylar notch. **c** Computed tomography axial view: posterior loose bodies and multiple intercondylar notch osteophytes

**Fig. 3:**
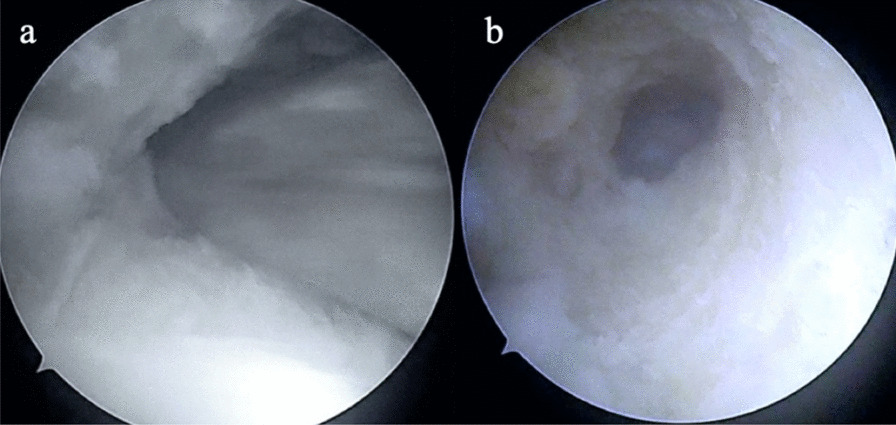
**a** Arthroscopic tunneling, using a shaver, through the intercondylar osteophytes, **b** intercondylar notch tunnel performed to allow the arthroscope to pass trans-notch

**Fig. 4:**
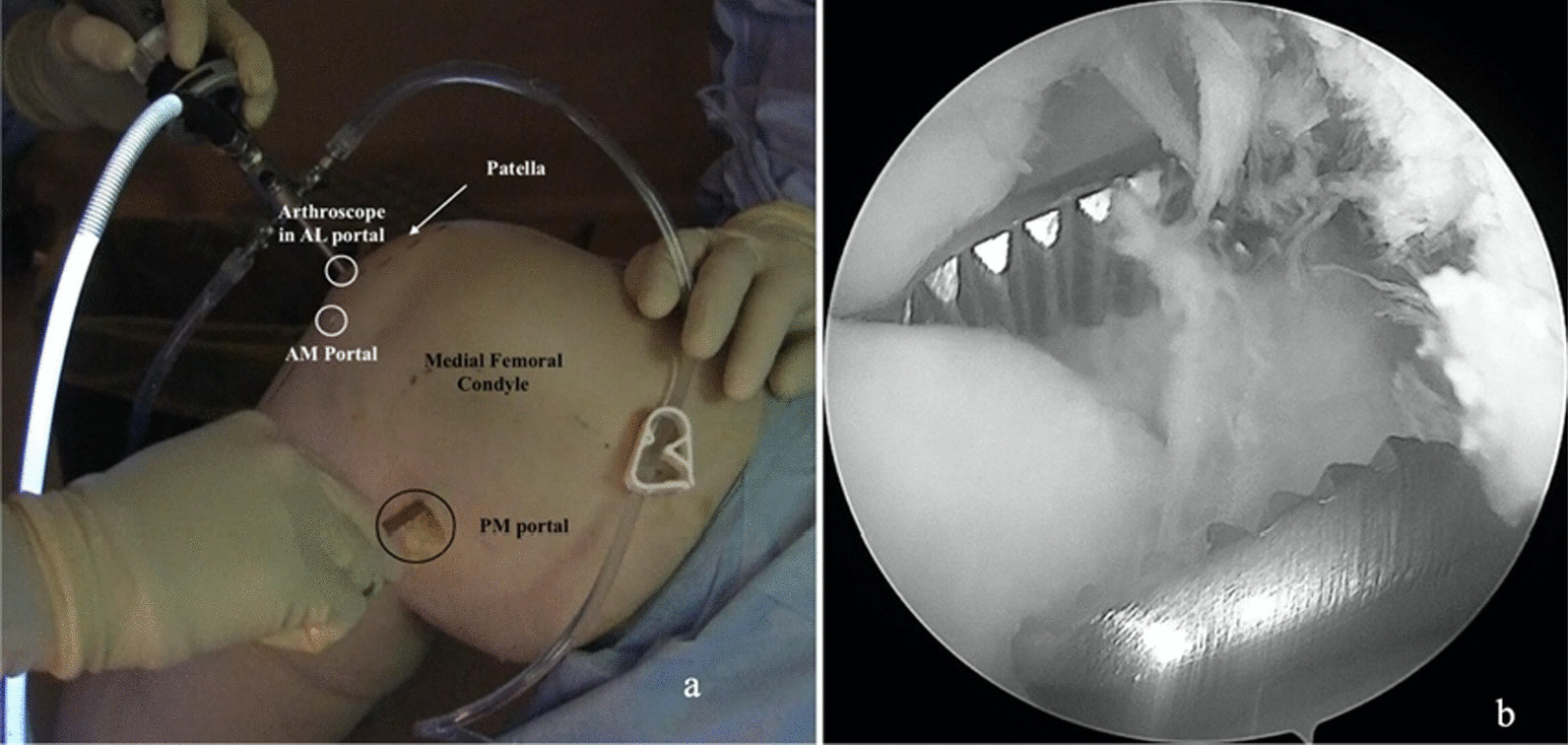
**a** Arthroscopic approach to the posteromedial compartment of the knee using a posteromedial (PM) portal. A 70° arthroscope from the anterolateral (AL) portal through the intercondylar tunnel, with the knee in 90° of flexion and forceps introduced through the PM portal. **b** Arthroscopic view of loose bodies removal using a 70° arthroscope

**Fig. 5:**
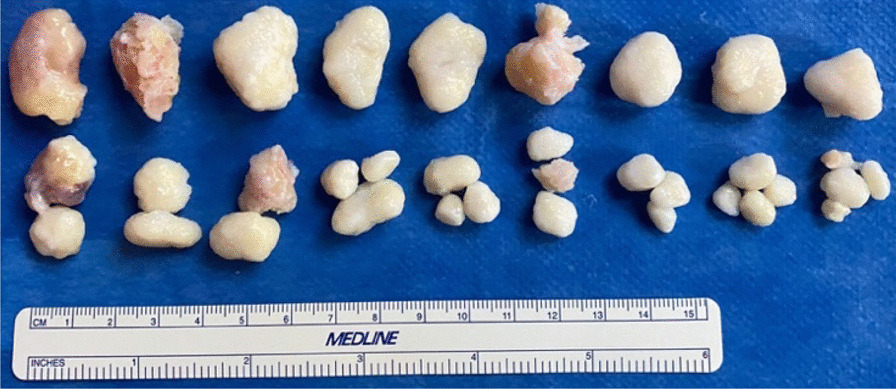
Loose bodies removed from the posteromedial compartment of the knee

**Fig. 6:**
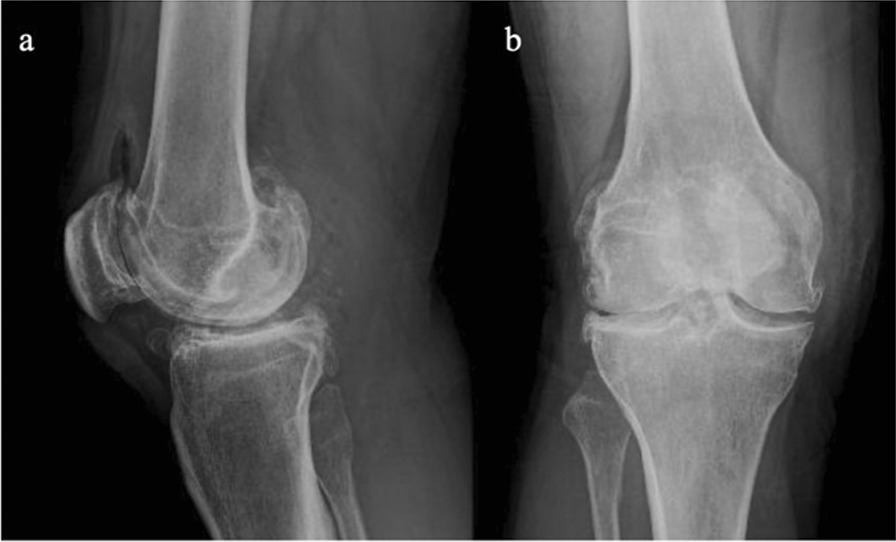
**a** Anterior–posterior and **b** lateral postoperative radiograph

**Fig. 7:**
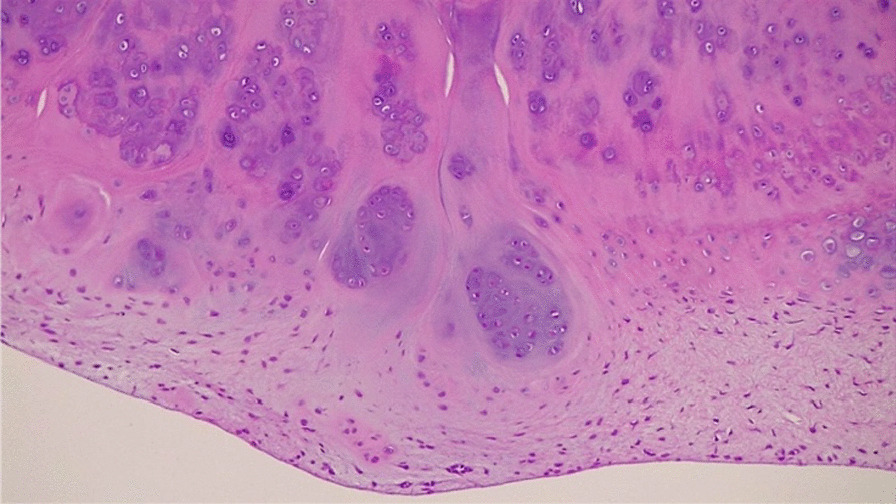
Histological examination of loose bodies, composed mainly of hyaline cartilage embedded in the connective tissue

A 35-year-old Caucasian male presented with discomfort and pain in the popliteal area of the right knee that had persisted for 11 years, without a history of trauma. He complained of pain progression from sporadic to continuous, intermittent swelling, and decreased ROM for the last 3 years. The medical history of the patient was unknown and not reported during the first medical examination. He reported he had undergone a surgical procedure to his knee because of the pain, 4 years before, without any significant relief of the symptoms. The surgical report documented an arthroscopic synovectomy combined with medial meniscus partial meniscectomy for a peripherical lesion through the standard anteromedial and anterolateral portals. After the surgery he continued to use painkillers, but no improvement of ROM has been registered. Our first physical examination revealed swollen knee, tenderness to palpation, decreased ROM (15–80°), and valgus limb alignment. Anterior–posterior and varus/valgus stress stability were preserved. No meniscal signs and focal neurologic deficits were reported. The symptoms were exacerbated upon deep flexion. On occasions, he could not walk or go up and down stairs owing to pain and articular locking. Weight-bearing radiographs showed advanced stage of osteoarthritis (Kellgren–Lawrence stage II–III), mild valgus limb alignment, and multiple loose bodies in the posterior compartment (Fig. 1a, b). In order to accurately localize the loose bodies in the posterior compartments of the knee, a MRI was performed. MRI scan is able to characterize synovial lesions, owing to their high resolution of soft tissue [25, 26] and non‐calcified cartilage nodules. It revealed diffuse chondropathy, minimal synovial hypertrophy areas, multiple osteophytes in the intercondylar notch, and more than 30 low density loose radio dense bodies in the posteromedial compartment. Owing to this, we classified the SC as stage III according to Milgram [7] (Fig. 2a, b). Considering the high grade of osteoarthrosis, we felt confident in performing a CT scan to better define the location of the osteophytes, the signs of osteoarthritis such as narrowing of the joint space and bone spurs, and the presence of calcifications and calcified loose bodies. In detail, the CT scan documented the bony structures, the morphology of the intercondylar notch, and the presence of osteophytes localized to the anterior and posterior side of the medial and lateral femoral condyles, allowing for a meticulous planning on how to reach the posterior compartment of the knee, passing anterior to posterior through a trans-notch passage (Fig. 2c). Even though the radiological exams showed an advanced grade of arthritis, the patient’s age and the desire for a full functional recovery drove us to perform a less invasive procedure than a total knee replacement, with the aim to preserve his native joint. For this reason we chose an arthroscopic procedure, considering that the large amount of loose bodies in the posterior knee might be the reason for the limited ROM and recurrent synovitis. The patient underwent an arthroscopic surgical approach 2 months after our first examination. Under spinal anesthesia, 2 g of intravenous cephalosporin was administered before inflation of a thigh tourniquet. The patient was placed supine on the operating table with the operative leg hanging in a 90° flexion position. A lateral post was placed just proximal to the knee at the level of the padded tourniquet, and a foot roll to prevent the hip from externally rotating and to maintain 90° of knee flexion. An arthroscopic examination of the anterior knee compartment was performed using conventional anteromedial (AM) and anterolateral (AL) portals, close to the patellar tendon border to facilitate passage of the arthroscope or instruments through the intercondylar notch. Multiple osteophytes were localized on the lateral side of medial femoral condyle, interfering with the trans-notch arthroscope passage. Meticulous debridement, removal of anterior osteophytes and a few anterior loose bodies that probably escaped from the posterior compartment, and tunneling, using a burr through the intercondylar osteophytes, were performed to allow the arthroscope to pass trans-notch (Fig. 3a, b). We performed the modified Gillquist maneuver [13] to reach the posteromedial knee compartment. The arthroscope from AL portal was introduced into the posteromedial compartment through the intercondylar notch, passing between the lateral border of the medial femoral condyle and the medial border of the posterior cruciate ligament, with the knee in 90° of flexion. The posteromedial (PM) portal was than created under the guidance of trans-illumination by the light source introduced into the AL portal, with the intent to prevent injury to the neurovascular structures. With the arthroscope in the AL portal, the forceps were introduced through the PM portal and all the loose bodies were removed. To avoid additional accessory posterior portals, we used a 70° arthroscope to better explore the posteromedial knee compartment (Fig. 4a, b). To complete the procedure, synovectomy of the inflamed areas was performed to remove the active synovial proliferative tissue. A total of 33 loose bodies were removed from the posteromedial compartment (Fig. 5). A final radiographic control documented the result of the procedure (Fig. 6a, b). Rehabilitation to recover ROM and full weight-bearing were permitted on postoperative day 1. Anti-thromboembolic prophylaxis, antibiotic prophylaxis, and pain killers were recommended. The patient was discharged on postoperative day 1. Histological examination showed that the loose bodies were composed mainly of hyaline cartilage embedded in the connective tissue, which confirmed the diagnosis of SC (Fig. 7). After 1 month, pain and swelling were limited. ROM was 5–90° and the anteroposterior and varus/valgus stability was maintained. After 3 months, the wound was completely healed without swelling of the knee. The patient reported pain only during high demanding activities and ROM was 0–110°. At 12 months of FU, the patient was pain free with a complete recovery of ROM, and was able to participate in some light sport activity. The Tegner Lysholm Knee scoring scale was excellent (95/100 points). No recurrence of swelling or locking symptoms were reported.

## Discussion

The localization of the loose bodies in the posterior compartment of the knee is an uncommon site for synovial chondromatosis. In presence of locking symptoms or decreased ROM, the gold standard treatment is surgery [3] but the choice of the proper surgical approach might not be easy for the surgeon. Other non-surgical treatment, such as chemotherapy and radiotherapy, usually have no role in the treatment of SC [5], despite the case reported by Chong *et al*., that demonstrated the effectiveness of radiotherapy for refractory knee SC [14]. The difficult location of loose bodies in a patient with severe osteoarthritis and multiple anterior osteophytes might encourage the surgeon to perform an open approach or a total knee replacement directly, despite young age. Furthermore, the challenging arthroscopic access might drive the surgeon, during the arthroscopy, to convert the procedure to an open approach in order to remove loose bodies from the posterior compartment [9]. In literature, few authors reported chondromatosis in the posterior compartment being successfully treated using different arthroscopic posterior portal approaches [9, 15]. The posteromedial, the trans-septal, and the posterolateral portals allow for the full exploration of the posterior compartment and to perform a meticulous debridement and loose bodies excision [12]. In our case we had the aim to report a difficult case of SC of posterior knee compartment in a patient with sever osteoarthrosis using the posteromedial portal. The decision to avoid a total knee arthroplasty was driven by the patient’s age and the association with a high complication rate when this procedure is performed in the setting of SC of the knee [16]. In literature, controversy remains over the optimal treatment when comparing the open approach with the arthroscopic procedure [12, 17]. Loose bodies are usually located at the posterior compartment owing to the gravity effect, and in many reports are found in the posteromedial compartment of the knee [18]. Some authors reported different ways for posteromedial visualization of the loose bodies [18–20], but no one challenged with a knee with osteoarthritis and anterior osteophytes made the trans-notch passage complex. In our experience, preoperative planning with MRI and CT scans was essential to predict the difficulties we might have found during the procedure. Furthermore, performing an appropriate tunneling of the intercondylar notch through osteophytes in the space between the PCL and medial femoral condyle was crucial to reach and visualize the posteromedial compartment of the knee through a trans-notch passage. Moreover, the use of a 70° arthroscope helped us to explore the posteromedial compartment well. The combination of these two techniques allowed us to perform an arthroscopic procedure without converting the surgery to an open approach. Avoiding the traditional open synovectomy, with extensive posterior approach and often combined with an arthroscopic anterior loose body removal, prevents complications such as persistent joint stiffness and prolonged rehabilitation [17]. An all-arthroscopic approach minimizes complications and reduces postoperative morbidity, providing adequate control of local disease, although it is technically demanding [18]. However, the effectiveness of synovectomy for SC remains controversial. Many studies reported that removal of loose bodies alone was preferable to synovectomy, and total synovectomy was not recommended because of risk of stiffness [2, 21, 22]. On the other hand, many authors reported that patients treated with synovectomy had lower recurrence rates [23–25] when compared with ones treated with isolated removal of bodies. To our knowledge, synovectomy has to be adopted in the case of inflamed foci of synovia. According to Urbach *et al*. [26], the removal of loose bodies combined with localized synovectomy to completely eliminate abnormal synovial tissue may decrease recurrence rate. To reduce the potential for recurrence, removal of loose bodies along with synovectomy has been recommended [23].

## Conclusion

Our case report highlights the importance of the arthroscopic approach to treat SC, despite the involvement of the posterior compartment of the knee. An optimal preoperative imaging allows for the planning of the proper surgical procedure, even in patients with severe osteoarthritis. The adoption of an intercondylar notch tunneling and a 70° arthroscope can help surgeons to better explore the posterior knee compartment, avoiding an accessory posterior trans-septal portal. Therefore, a synovectomy of the inflamed foci may be useful in preventing recurrence. The patient,who had severe osteoarthritis, has recovered good knee function, returning to his basic activities of daily living and sport activity, while preserving his native joint.

## Data Availability

The dataset supporting the conclusions of this article is included within the article.

## Abbreviations

SC: Synovial chondromatosis
CT: Computed Tomography
MRI: Magnetic Resonance Imaging
ROM: Range of motion
AM: Anteromedial
AL: Anterolateral
FU: Follow-up
